# Physical Activity and Bone Health: What Is the Role of Immune System? A Narrative Review of the Third Way

**DOI:** 10.3389/fendo.2019.00060

**Published:** 2019-02-07

**Authors:** Giovanni Lombardi, Ewa Ziemann, Giuseppe Banfi

**Affiliations:** ^1^Laboratory of Experimental Biochemistry & Molecular Biology, IRCCS Istituto Ortopedico Galeazzi, Milan, Italy; ^2^Department of Physiology and Pharmacology, Faculty of Rehabilitation and Kinesiology, Gdansk University of Physical Education and Sport, Gdansk, Poland; ^3^Vita-Salute San Raffaele University, Milan, Italy

**Keywords:** exercise, training, biomechanical loading, inflammatory mediators, inflammasomes

## Abstract

Bone tissue can be seen as a physiological hub of several stimuli of different origin (e.g., dietary, endocrine, nervous, immune, skeletal muscle traction, biomechanical load). Their integration, at the bone level, results in: (i) changes in mineral and protein composition and microarchitecture and, consequently, in shape and strength; (ii) modulation of calcium and phosphorous release into the bloodstream, (iii) expression and release of hormones and mediators able to communicate the current bone status to the rest of the body. Different stimuli are able to act on either one or, as usual, more levels. Physical activity is the key stimulus for bone metabolism acting in two ways: through the biomechanical load which resolves into a direct stimulation of the segment(s) involved and through an indirect load mediated by muscle traction onto the bone, which is the main physiological stimulus for bone formation, and the endocrine stimulation which causes homeostatic adaptation. The third way, in which physical activity is able to modify bone functions, passes through the immune system. It is known that immune function is modulated by physical activity; however, two recent insights have shed new light on this modulation. The first relies on the discovery of inflammasomes, receptors/sensors of the innate immunity that regulate caspase-1 activation and are, hence, the tissue triggers of inflammation in response to infections and/or stressors. The second relies on the ability of certain tissues, and particularly skeletal muscle and adipose tissue, to synthesize and secrete mediators (namely, myokines and adipokines) able to affect, profoundly, the immune function. Physical activity is known to act on both these mechanisms and, hence, its effects on bone are also mediated by the immune system activation. Indeed, that immune system and bone are tightly connected and inflammation is pivotal in determining the bone metabolic status is well-known. The aim of this narrative review is to give a complete view of the exercise-dependent immune system-mediated effects on bone metabolism and function.

## Introduction

Exercise, particularly when energetically demanding and sustained, affects all human tissues and organs. Different kinds of exercise (e.g., endurance, high intensity, resistance) have different effects on tissues and organs homeostasis and, consequently, different kinds of training require different adaptive changes that might take place (1). Bone is importantly affected by exercise and bone cells metabolism forcefully adapts to training. This metabolic adaptation reflects, on long-term basis, in a micro-architectural, and possibly macro-architectural, redefinition of bone shape and structure. The biological meaning of this phenomena resides in the physical adaptation of bones (in terms of shape, mass, and strength) to the changed environmental conditions (applied forces) (2). However, the bony response to exercise does not only depend on the biomechanical stimulus but equally relies on other systemic mechanisms that make the bone a center for the integration of the signals generated during and following the exercise (3). A main signal is the metabolic one: bone metabolism is highly-demanding in terms of energy; parallel, during exercise the energy needs increase with the increasing duration and intensity. Being not a lifesaving function, bone metabolism is partially blocked in response to acute exercise, regardless the degree of biomechanical load. This block mainly pertains the anabolic function (osteoblastic function, i.e., bone formation) while leaves almost unaltered the catabolic side (osteoclastic function, i.e., bone resorption). The acute unbalance toward bone resorption makes the calcium, stored in the bone matrix, available to skeletal muscles (SKM) and cardiac muscle for contraction and to the brain to sustain the neuromuscular function (4). On the other hand, the chronically established bone metabolic response to training is driven by the degree of load: weight-bearing/high impact activities (e.g., plyometric) shift the balance toward bone formation and on average increase bone mass and bone mineral density (BMD). Equally demanding activities featured by a low/absent biomechanical load (e.g., swimming, cycling) shift the balance toward resorption and causes a decrease in the average bone mass and BMD (3). Another key signal, generated during and following exercise and affecting bone metabolism, is the immune/inflammatory one. Inflammation, acute and chronic, is a main determinant for bone metabolism and the plethora of inflammatory mediators, produced under either physiologic or pathologic conditions, affects bone cells. Noteworthy, in last 15 years an interdisciplinary branch of study embracing (but not limited to) endocrinology, immunology, orthopedics, and rheumatology, namely osteoimmunology, has developed quickly thus becoming a central subject in metabolic diseases of bone (5). A key role of the osteoimmune networking has been highlighted by the clinical success and safety, over the classical anti-resorptive drugs (6, 7), of the fully human monoclonal antibody denosumab used in the treatment of primary and secondary osteoporosis, in both males and females (8), which is an inhibitor of the prototypic osteoimmunological signaling pathway, the RANK/RANKL system, as described elsewhere (9). Acute exercise represents a powerful inflammatory stimulus itself while the inflammatory response to training is a handbook example of adaptation to a continuous stimulus. Exercise, indeed, initiates a series of inflammatory events, which ultimately, if chronically continued, positively affects health. The inflammatory response to exercise, however, takes place in the immune system (IS), involving mainly the innate compartment with fallouts on the humoral immunity, as well as in tissues other than the IS, e.g., SKM, white and brown adipose tissues (WAT, BAT), brain, liver (3, 10, 11). Notably, the acute response elicited by exercise in terms of inflammatory mediators is often very similar to that observed during pathological acute and chronic inflammatory conditions (e.g., sepsis, obesity, autoimmune diseases, tumors) but what determines the net result of this response (pro- vs. anti-inflammatory) is the milieu in which this response is generated, together with its temporal length and the site of production. As an example, interleukin (IL)-6 expression is strongly induced by time, intensity and type of muscle contractions-dependently as well as in sepsis (various origin) but, in the latter case, it is driven by the previous increase in another cytokine, the tumor necrosis factor (TNF)-α (12). Based on the interdependence between bone and IS, it is implicit that the acute inflammatory response induced by exercise- and the modulation of the inflammatory status induced by chronic training both affect bone cells differentiation and function and, in turn, the bone metabolism. It should be kept in mind that, other than a target of these endocrine and inflammatory stimuli, the bone is itself a source of these mediators and, hence, it actively enters in the regulation/modulation network of the homeostasis (3).

This review aims at summarizing the experimental evidences about the exercise- and training-dependent effects on bone mediated by the IS and the other inflammatory sources in order to depict additional, and less explored, link between bone and inflammation as further mechanisms by which the physically active status is a main determinant for health.

In order to go deep into the description, it is necessary to introduce some key concepts, relative to the exercise physiology, that will help the reader in fully understand the biological meaning underlying the homeostatic response. About the types of muscle actions, one can distinguish between static (isometric) and dynamic (isotonic) actions. During the static (isometric) action, muscles generate force without changing length due to an external resistance (weight of an object) greater than the force produced by the muscle; despite the energy expenditure, no work is done due to lack of movement. The isotonic action, instead, can be either (i) concentric, when muscles produce enough force to overcome the external resistance and the muscle contraction results in a work, or (ii) eccentric, when the muscles lengthens while generate a force due to the opposite movement of the external resistance and the sarcomere shortening (13). Another series of concepts regard the terms physical activity (PA), exercise, and training. PA is defined as any bodily movement produced by SKM resulting in an energy consumption. Everyone performs PA in order to sustain life but the amount is subject to personal. The term “exercise,” instead, although often erroneously used interchangeably with PA, is defined as a PA featured by planning, structure, and repetitiveness, which is aimed at maintaining or improving the physical fitness (14). The common and distinctive features of PA and exercise are summarized in Table 1.

**Table 1:** Common and distinctive features of physical activity and exercise.

**Physical Activity**	**Exercise**
Body movement generated by skeletal muscles Variable energy expenditure Positive correlation with physical fitness	Body movement generated by skeletal muscles Variable energy expenditure Positive correlation with physical fitness Planned, structured, and repetitive Aimed at maintain/improve physical fitness

When a single bout of exercise (acute exercise) is continued over the time, in the same fashion, it is defined training (exercise training). Finally, the different types of exercise and training can be categorized as follows: (i) endurance, mainly based on the aerobic metabolism (e.g., distance running, road cycling, swimming, triathlon), (ii) resistance (also known as strength), mainly based on the anaerobic metabolism (e.g., weight lifting, discus, hammer, and javelin throw) (15).

## How Do Exercise and Training Affect Bone Metabolism?

The responsiveness of bone to mechanical stimulation was first theorized by Frost who postulated, with the “mechanostat” hypothesis, bone mass and structure remain constant around a certain threshold of mechanical strains. Bone formation takes place when the strain increases above this threshold, and it results in an increased bone stiffness. When the strain experienced by the bone segment is lower than this threshold bone loss can take place (16). Later, it was shown that the threshold itself is modifiable by several factors, mainly endocrine [parathyroid hormone (PTH), sex hormones, etc.] (17). However, despite its importance, the mechanical strains induced by strenuous PA is very small degree attesting to up to 0.3% (3,000 microstrain) (18). Based on that, it is likely that bone cells are exposed to and integrate different PA-generated mechanical stimuli that altogether imply an amplification of the environmental stimulation. A further level of complexity is due to the fact that different types of bone cells are anatomically exposed to different combinations of stimuli. Bone marrow and endosteal osteoblasts experience the pressure forces generated within the marrow cavity. Osteocytes buried into the matrix with their interconnecting long cellular processes running within the fluid-filled canalicular network experience dynamic fluid flow pressure, shear stress forces, and dynamic electric fields (due to the transit of charged ions in the interstitial fluid). Mature osteoclasts and their precursors, residing in the bone marrow, may be exposed to mechanical stimulation due to dynamic pressure (19). Bone mechanosensitivity is mediated by several cellular components (e.g., membrane, membrane proteins, cytoskeleton, cilia, ion channels). Shear stress and pressure deform the plasma membrane and, consequently, to the cytoskeleton and, in turn, through integrins to the protein machinery mediating the cell-to-matrix adhesion and to the nucleus where it induces the expression of downstream genes (20). In osteoblast, the deformation of the plasma membrane is associated with the activation of ion channels (21), as in osteocytes, whose cilia, protruding out of the dendritic extensions, sense fluid flow and activate channel-mediated ion fluxes that modulate the Wnt signaling pathway (22).

The different nature of the mechanical stimuli together with the number of cell structures involved in mechanosensitivity imply the integration of the different signals generated (19). Indeed, the physical stimulus is translated into several chemical signals including calcium, mitogen-activated protein kinase (MAPK), Wnt, and RhoA/ROCK pathways. For instance, the Frizzled-LRP5/6-mediated activation of Wnt induces the expression of osteoblastic factors, as RUNX2 that promotes the commitment of mesenchymal stem cells (MSCs) toward the osteoblast lineage, induces proliferation and differentiation of pre-osteoblast, and stimulates mineralization. Hence, exercise shifts the adipogenic-to-osteogenic equilibrium, governing the MSC fate, toward the osteoblastic commitment (23, 24). By modulating the OPG and RANKL expression in osteoblasts, Wnt signaling also downregulates osteoclastogenesis and osteoclast activity (25). A key mechanism regulating the Wnt activity that underlies the exercise-associated effects on bone is mediated by sclerostin (Sost) (26). Osteocytes constitutively produce Sost that inhibits the Wnt pathway, thereby osteoblastogenesis and bone formation. Loading activates a molecular response that inhibits Sost expression and, then, allow the activation of Wnt signaling. Noteworthy, also prostaglandin E2 (PGE2), which is induced by strain sensing, by activating its receptors EP2 and EP4, facilitates the nuclear translocation of β-catenin and its transcriptional action (27). Finally, the intracellular signaling generated following mechanical stimulation can be propagated to neighbor cells as calcium (e.g., between osteoblasts and osteocytes) or adenosine triphosphate (ATP; e.g., osteoblast-to-osteoclast) transients through the gap junction network (28, 29).

Another way by which exercise and recovery beneficially affects bone metabolism, mineral content, and structure is by increasing the blood flow to the bone and the consequent improved supply of nutrients supporting its metabolic needs (30, 31). Indeed, also from this point of view, bone is a really active tissue as demonstrated by the fact that blood flow to the bone and glucose uptake within the bone tissue are increased in response to exercise (32). However, as exercise load increases blood flow offs due to the sympathetic response that shifts the flow to the active SKM. Moreover, perfusion in bone, which ranges over a window wider than that expected during exercise, is mainly ruled by chemoreceptors, and hence nutrients, rather than by hypoxia (33). The exercise-induced enhanced bone perfusion also causes an increased efflux of stem cells from bone marrow (e.g., endothelial precursors) (34), consequently to the endothelial production of nitric oxide (35) and sympathetic nervous system activation (36). Finally, exercise-induced muscular-derived adenosine could solve another key role in bone blood flow determination (37).

### The Effect of Acute Exercise

Despite the key role of loading, the bone tissue response of the to a single bout of exercise is mainly driven by the exercise-dependent metabolic requests of noble organs and of those organs that are directly involved in the activity. In other terms, during PA, and mostly dependent upon its intensity and duration, the metabolic needs of non-life saving organs (e.g., the bone, skin, gut) are somehow “sacrificed” in order to have all the fuels available for being used by brain, SKM, and liver (3). After the conclusion of the PA, during recovery, and with the chronic and/or long-term repetition of the act, the loading/impact-induced anabolic response takes place. This means that during PA the bone metabolism is mostly unbalanced toward resorption. This is also a consequence of the key role of bone in calcium homeostasis: PA implies the usage of calcium in terms of functioning of the contractile machinery of the SKM, release, and recycling of neurotransmitters and hormones. Being resorbed, bone make the calcium promptly available. Indeed, two activities both characterized by high energy expenditures but differentiated by kind of biomechanical loading, one featured by high load and impact (mountain ultra-trail) while the other non-impact/loading (cycling), both display an anti-anabolic/pro-catabolic acute response of the bone to the activity (38, 39).

By considering the biomechanical aspects, only dynamic stimuli can generate an osteogenic response that, instead, is not induced by static loading (40) and strain amplitude, number of cycles and interval between the cycles are important, as well (41, 42). Indeed, the anabolic response can be desensitized by long-lasting activities while interval rest between the load cycles may have an osteogenic effect (43, 44). On the contrary, inter-cycles resting activates some mechanotransduction-sensitizing mechanisms that sustain the osteogenic response (28). However, too high magnitudes or number of cycles of load may fatigue the bone tissue and consequently can cause microdamage that, in turns, result in a series of catabolic events including local bone resorption (45).

### The Effect of Chronic Exercise and Training

Life-long PA is associated with a better bone quality, thus potentially resulting in a stronger bone, e.g., improved cross-sectional area, BMD, and moments of inertia. These features, for example, and have been observed in gymnasts vs. non-gymnasts (46, 47), and dominant vs. non-dominant limbs of racquet sports players (48) or triple jumpers (49). By applying the same external force, the deformation experienced by “weak bones” is greater than that of “strong bones” and, consequently, it elicits in larger tissue strains. This results in a greater anabolic response in the weaker bone that attempts to become stronger (50). In experimental models, loads causing high strains induce bone formation in loaded areas, while areas with lower peak strains featured by reduced bone formation or even increased bone catabolism (51). Site-specific adaptation to loads takes also place in human: in women, after skeletal maturity, the adaptation to load is related to the energy equivalent strain, which means that regions undergone to high-level strain experience bone apposition than regions undergone to low-level strain (50).

However, the bone adaptation to chronic PA and training mainly depends upon the kind of activity. This was clearly demonstrated by Nikander et al. (52), who evaluate the bone quality of the narrowest section of the femoral neck [areal BMD (aBMD), hip structure analysis (HSA), cross-sectional area (CSA), subperiosteal width (W), and section modulus of strength (Z)] in 225 premenopausal women performing different sports. Women performing high-impact (volleyball, hurdling) and odd-impact (squash, soccer, speed skating, step-aerobic) loading sports displayed the highest aBMD (+23 and +29% vs. non-athletic women), CSA (+22 and +27%), and Z (+22 and +26%) even following adjustment for age-, weight-, and height. Contrarily, low-impact (orienteering, cross-country skiing) and non-impact (cycling, swimming) repetitive loading activities were associated with no gains in bone quality, compared with the inactive controls (52). A Cochrane review evaluating the preventive and therapeutic effects of training on postmenopausal osteoporosis established that exercise has a small (about 3%) but significant effect on bone mass and BMD. According to the meta-analysis, femoral bone mass mostly benefits from high-force non- weight-bearing exercises (e.g., resistance strength training of the lower limbs). Vertebral bone mass, instead, mostly benefits from the combination of exercises featured by different types of dynamic loading. Consequently, the risk of fracture across all exercise groups was not significantly different compared to the control groups (53). Exercise-based, either preventing or therapeutic, strategies aimed at affecting bone health might account for this finding. Indeed, based on our recent overview of systematic reviews and meta-analyses, lifelong age-specific exercise is effective in sustaining bone health in women. School-based short bouts of high-impact plyometric exercises positively affect peak bone mass in young girls, while combined-impact exercises represent the best exercise modality to preserve/improve BMD in both pre- and post-menopausal women (54).

Beside the quite well-depicted effects of chronic exercising on the physical feature of the bone, the effects on blood flow or metabolism of human bone are scarce (33). Although neglected, these studies are of key importance since they highlight a role for bone in regulating the whole-body metabolism (55–57) and also because the bone is central in the release of vascular precursor and immune cells. Thus, it emerges that by influencing bone status, exercise training could potentially affect vascular impairments in pathological conditions such as diabetes (33) and it may regulate the release of immune cells into the circulation and, hence, to directly control the inflammatory status.

As important as loading, or even more important, is detraining. Constant load (use of the skeleton) is essential for osteocyte survival and in case of bone immobilization osteocyte apoptosis occurs (58). Parallel, detraining causes bone loss and, hence, training might be continued to maintain bone mass (59) since unloading and disuse increase bone resorption rate. This situation is encountered, for instance, in astronauts under weightlessness conditions, spinal cord injured patients, and elderly people forced to either partial or total immobilization (19). Unloading affects both the cortical and trabecular portions of the bone: spinal cord injured subjects experience a 20–40% decrease in cortical thickness (60) and an average 14% (range 2–80%) in trabecular density (61); for astronauts the mean BMD loss of the trabecular compartment was 4% at the lumbar spine and 12% at the proximal femur (62).

## How do Exercise and Training Affect the Inflammatory Response and the Immune Function?

Exercise profoundly affects the normal functioning of the IS with immune responses to single bouts being transient while an immune adaptation is likely to take place with training. Exercise dose is important in determining the entity of the immune response: prolonged intense training can have depressive effects (e.g., increased infection risk), while regular moderate-intensity exercise has more balanced effects that mainly results in the improvement of the baseline immune reactivity (63). Several evidences support this effect, chronic exercise has been demonstrated to improve immune, and hence health and behavioral outcomes, in several conditions of deregulated immune response, such as aging, obesity, cancer, and chronic viral infections (e.g., HIV) as well as in preventing their onset (63–66). Interestingly, acute exercise is effective in improving vaccines response (63). These effects are mediated, from one hand, by the cells of the IS belonging to both the innate and adaptive branches and, from the other hand, by all the cells of the body that are induced to express either a pro-inflammatory or anti-inflammatory phenotype. Exercise activates inflammatory cascades involving cells belonging to both the innate and adaptive immunity branches, cytokines, and mediators with active roles in inflammation (myokines, adipokines) that are in turn responsible for the generation of an environment in which recovery, regeneration, and adaptation take place. Exercise duration, mode, and intensity are the determinants for the exercise-induced inflammatory response. At the same time, training exerts anti-inflammatory actions through several distinct mechanisms involving metabolic, endocrine, and immune mediators of various tissues and organs (67). However, exercise exerts its anti-inflammatory effect only after the activation of pro-inflammatory cascades (67).

A special mention might be spent about cortisol. Although this adrenal hormone has anti-inflammatory immunosuppressant actions, and its occasionally increased circulating concentration (e.g., following PA) are beneficial in reducing inflammation, its chronically high levels (i.e., hypercortisolism) and deregulated rhythms are associated with aging and age-associated low-grade inflammation (68). Cortisol and the other endogenous glucocorticoids (GCs) are the final products of the neuroendocrine hypothalamus-pituitary-adrenal cortex (HPA) axis that is responsible for the regulation of both the energy balance and stress response. The physiological circadian fluctuations of GC levels allow the correct functioning of the intermediate metabolism and the development and the maintenance of the homeostasis of a wide all the body tissues, including the bone. Indeed, GCs are essential for bone modeling and remodeling as they promote osteoblastogenesis to maintain the bone architecture (69). Excessive energy intake (unbalanced energy intake-to-energy expenditure ratio) and adiposity are associated with chronic inflammation and stress which are in turn responsible for a deregulated of the HPA axis. Moreover, hypercortisolism is associated with a deregulated energy metabolism that in turn is responsible for the maintenance of the chronic inflammatory state (70). PA, by acting as a chronoenhancer, also impacting on the HPA axis, is able to improve the GC response in healthy subjects as well as to restore the circadian rhythm in age-associated low-grade inflammation dependent hypercortisolism and, hence, to improve the related comorbidities (68).

### Effects of Exercise and Training on Inflammation

#### Effect of Acute Exercise

A single exercise bout starts a series of timely-defined inflammatory events, which mainly depend upon mode, intensity, duration, and training status (i.e., familiarity with the exercise). This cascade starts with a pro-inflammatory phase (1.5–24 h post-exercise) which is then followed by an anti-inflammatory phase that sustains SKM regeneration (24–72 h post-exercise). The exercise-induced inflammatory response is evidenced by the rise of the circulating levels of myokines (i.e., IL-6) and anti-inflammatory mediators (IL-10, IL-1ra). Moreover, exercise downregulates the expression, on the surface of antigen-presenting cells (APCs, e.g., monocytes) of those receptors involved in the recognition of danger signals, i.e., toll-like receptors (TLRs) (67). TLRs are highly evolutionarily conserved transmembrane proteins involved in the recognition of classes of molecules non-specifically associated with pathogens (pathogen-associated molecular patterns, PAMPs) and “danger signals” non-specifically induced/released following tissue damage due to physical, chemical, or biological agents (danger-associated molecular patterns, DAMPs) (71). TLRs activation leads to the expression of inflammatory cytokines. Acute exercise affects the expression of TLRs on monocytes and, hence, by desensitizing these cells to pro-inflammatory stimulation, this results in a push toward the anti-inflammatory phenotype. 2.5 h of cycling at 60% of VO_2_max induces a significant decrease in the expression of TLRs on CD14+ monocytes, compared to rest, immediately (TLR2, about −25%) and 1 h post-exercise (TLR1, −60%, TLR2 and TLR4, −50%, TLR3, −30%) (72).

#### Effect of Chronic Exercise and Training

There are several potential tissue-specific anti-inflammatory mechanisms associated with regular PA and these include reductions in body fat (particularly, visceral fat), enhanced expression and release of contracting muscle-derived anti-inflammatory mediators, downregulated expression of TLRs in monocytes and macrophages, increased expression of anti-oxidant species counteracting the exercise-associated rise in reactive oxygen species (ROS) generation (73).

Exercise causes transient elevations in IL-6 coming from exercising SKM (74). Contrarily, during inflammation, and especially chronic low-grade inflammation, IL-6 is produced in a slightly chronically elevated manner and, in this case, the source is represented by immune cells and hepatocytes (3). Muscle-derived IL-6 has anti-inflammatory effects by inducing other anti-inflammatory cytokines (e.g., IL-1ra and IL-10) that antagonize the pro-inflammatory IL-1β and TNF-α (12). IL-6, but also the exercise-related increased energy needs, stimulate the release of cortisol that has an immunosuppressant activity (74). The logic around the IL-6 release by the contracting muscle resides in its activity aimed at increasing the usage and delivery of energetic substrates to the myocytes, in concert with the stress hormones (e.g., cortisol, epinephrine). By acting in autocrine and paracrine fashions, indeed, IL-6 stimulates the cellular uptake and the oxidation of glucose and fatty acids by the SKM itself; contemporary, by acting in an endocrine fashion, it induces lipolysis at the AT level and glycogenolysis and gluconeogenesis. The insulin-mimetic effect of IL-6 on glucose uptake is of particular interest. The binding of IL-6 with the IL-6Rα/gp130Rβ receptor complex leads to the activation of AMPK that triggers the plasma membrane translocation of intracellular vesicles bearing the insulin-dependent glucose transporter (GLUT4) allowing glucose uptake regardless the insulin status. This accounts for the beneficial effects of PA on the metabolic function also in impaired glucose tolerance and insulin resistance (75). Moreover, other than stimulating the release of IL-6, exercise training generates an appropriate environment making the IL-6 effect distinctly anti-inflammatory: for instance, aerobic training reduces the expression of TNF-α and IL-1α by mononuclear cells and induces IL-4, IL-10, and TGF-β1 in subjects at high-risks of heart disease (76).

Training exerts its effects by also reducing the activation potential of the innate immune response activation in terms of TLRs in an age-independent manner. Young (18–35 years of age) and elderly (65–80 years of age) active subjects have a one third reduced expression of TLR4 on CD14+ monocytes' surface, compared to their inactive counterparts. Moreover, 12-week of either endurance or resistance training halved TLR4 expression in monocytes from these inactive (old and young) subjects to a level comparable to those found in active age-matched controls, while the intervention was ineffective in the already active subjects (72).

The anti-inflammatory effect of training might is also the result of the modulation of nitric oxide (NO) and ROS production and the consequent activation of their downstream pathways. Exercise induces the synthesis of NO and ROS which are important in inducing anti-inflammatory defensive mechanisms especially by targeting muscle gene expression (77). In the case of ROS, with training, the cyclic exercise-induced spiked production (contrarily to what happens in chronic inflammation) causes the activation of an adaptive response that, in turn, protects SKM from exposure to the exercise-dependent increase of ROS itself. This phenomenon accounts also for the decreased expression of TNFα that may further inflammation (77).

The AT is determinant in defining the inflammatory status. The association of physical inactivity and high caloric intake results in adipocyte and AT hypertrophy. As adipocytes grow, the oxygen supply becomes limiting and the consequent hypoxic stress leads to cell death and necrosis. Necrosis recalls macrophages and potently induces an inflammatory response (78). Notably, this process seems to involve mainly the visceral AT that has a higher inflammatory potential than the subcutaneous one. Exercise-induced caloric imbalance causes lipolysis, aimed at mobilizing fats to be used as fuel by the exercising muscle, with a reduction of the adipocytes' size and, thereby, hypoxic stress and inflammation (79).

## Effects of Exercise and Training on Immune Functions

Exercise profoundly affects the IS functioning. An exercise bout causes an important redistribution of leukocytes as a consequence of the hemodynamic response and the increased blood levels of catecholamine and glucocorticoids, but the effects depend upon exercise intensity and duration. Prolonged periods of intensive training can impair immune functions, and particularly those of T-cells, natural killer (NK)-cells, and neutrophils; in elite athletes, during periods of heavy training and competition, mucosal immunity is also affected determining an increased risk of infections of the upper respiratory tract. Contrarily, regular moderate-intensity activities are beneficial having immune-enhancing effects and, as stated above, throughout the reductions of inflammation, increased immune cells turnover, enhanced immune surveillance, and improvement of psychological stress status (63).

### Effect of Acute Exercise

An exercise bout increases both the absolute and relative leukocyte counts. Transient leucocytosis takes place already after brief (minutes) dynamic exercise and is more sustained in the case of prolonged endurance exercise (80) and returns to pre-exercise levels within 6–24 h (63). Neutrophils and lymphocytes are mainly involved in this response while a smaller contribute is given by monocytes. The response, however, differs for these cells: during the early phase of recovery (30–60 min after exercise), the neutrophilia is associated with lymphocytopenia that can be particularly pronounced until clinically relevant low levels (<1.0·10^9^/L, which means −30 to −50% compared to pre-exercise values) and can last up to 6 h (80). Other features also characterize this response. Exercise tends to mobilize cytotoxic cells (e.g., NK-cells, and CD8+ and γδ T-cells) (81) and non-lymphocyte effector cells (e.g., CD16+ monocytes and CD16- neutrophils) (82, 83). The exercise-dependent mobilization mainly involves those cells with a higher migration potential, e.g., leukocytes expressing high levels of integrins and intracellular adhesion molecules (84) and a wide range of chemokine receptors (85). Finally, in these leukocytes the expression of adrenoreceptors (β2-ARs) and glucocorticoid receptors is upregulated, and are therefore they are highly responsive to catecholamines and cortisol (81, 86, 87), this indicates that leukocyte trafficking between the blood and tissues is strongly influenced by both the sympathetic branch of the nervous system and the HPA axis activation.

Other than being dislodged from liver, lung, and spleen endothelia due to the exercise-induced increased blood pressure- and cardiac output-mediated shear stress (80), leucocytes come from lymph nodes, intestines, bone marrow, thymus, and SKM that contain large numbers of white cells. The contribution of primary (i.e., bone marrow, thymus) and some secondary (i.e., lymph nodes), lymphoid organs to the initial exercise-induced leucocytosis is limited, since their content in mature/differentiated cells. The bone marrow likely sustains neutrophilia during recovery from prolonged exercise, while lymph nodes and thymus mainly sustain the restoration of the blood lymphocyte count following the transient lymphocytopenia (63).

About innate immunity, submaximal exercises enhance neutrophil chemotaxis (88), phagocytosis (89), and spontaneous degranulation (90). Also the neutrophil oxidative burst is affected by the exercise although in an intensity-and duration-dependent fashion: cycling at 50 and 80% of VO_2_max have enhancing and impairing effects, respectively; moreover, during recovery from moderate-intensity exercise the oxidative burst is enhanced while it is impaired following exhaustive and prolonged activities (91, 92). Also NK-cell cytotoxicity is quickly induced by exercise but this response is followed by a delayed suppression during recovery but this likely mirroring changes in their number (93).

Adaptive immunity appears to be both augmented and inhibited depending on intensity, duration, and modality of exercise (63). In trained triathletes, following a half-Ironman race, an intradermal inoculation containing several recall antigens caused a reduced 48 h-delayed-type compared to both resting triathletes and moderately trained healthy men (94).

### Effect of Chronic Exercise and Training

In general, high-intensity and high-volume training is thought to cause short- or long-term immune depressive states that can increase infection risk. Repeated bouts of strenuous exercise, performed without adequate recovery, result in a chronic state of impaired immunity (80). The decline in the count of circulating immune cells is associated with the increased susceptibility to infections: although athletes and healthy age-matched controls have comparable absolute and relative leukocyte counts, endurance athletes may experience reduced resting lymphocyte (runners) and NK-cell (swimmers, cyclists) counts (95–97). Functional declines in adaptive immunity associated with prolonged intensive training are related to unbalanced expression of pro- and anti-inflammatory cytokines and increased plasma levels of stress hormone (e.g., cortisol) (98).

Contrary to the strenuous, exhaustive exercise typically practiced by athletes, moderate-intensity training has beneficial effects on immune function (99). Moreover, exercise mode (i.e., aerobic, resistance, or combined) as well as the condition on which the intervention is addressed are main determinants of the immune effects. Indeed, moderate-intensity exercise training associates with a life-long improvement/maintenance of several aspects of the immune function (99, 100), such as increased response to vaccine (101, 102), viral infections (103, 104), and tumors (105–107), enhanced neutrophil phagocytic activity (108), T-cell proliferation (93, 109), NK-cell cytotoxic activity (93, 110), basal level of cytokines (111) and IL-2 production (112) and decreased number of senescent T-cells (113) and inflammatory response to bacterial challenge (114).

The enhanced adaptive immune response is also sustained by the improvement in systemic low-grade inflammation, driven by the improved inflammatory status of the AT, and hence in the associated adipokine profile (described above). Indeed, the downregulation of TLRs on the surface of monocytes (115) together with the direct exercise-induced M1 (pro-inflammatory)-to-M2 (anti-inflammatory) shift in macrophage phenotype, reduces the infiltration of the AT and, hence, its inflammatory status (116). The stress-related hormones released during exercise that have anti-inflammatory properties are responsible of a further stimulus: cortisol acting as an immunomodulatory and immunosuppressant compound and adrenaline that downregulates the expression of the inflammatory mediators IL-1β and TNF (73, 117). Exercise also decreases the content of cholesterol of the cell membranes that may improve T-cell receptor signaling and the translocation of MHC molecules for antigen presentation (118).

## Immuno-Mediated Effects of Exercise and Training on Bone

### Inflammasome Activation and Bone Metabolism

The innate immune function depends upon the recognition, by germline-encoded pattern-recognition receptors (PRRs), of PAMPs, derived from invading pathogens, and DAMPs, induced by endogenous stresses. PAMPs-/DAMPs-dependent activation of PRRs triggers the downstream signaling cascades and induces the expression of type I interferons (IFN-α, IFN-β) and pro-inflammatory cytokines (119). Inflammasomes are multimeric protein complexes assembling within the cytosol after sensing PAMPs or DAMPs (120, 121). They serve as scaffolds to recruit the inactive zymogen pro-caspase-1 that oligomerizes allowing the auto-proteolytic cleavage into active caspase-1. Active caspase-1 cleaves the precursor cytokines pro-IL-1β and pro-IL-18 generating the biologically active forms (122–124). Furthermore, when activated, caspase-1 can activate a series of intracellular events that lead to a form of cell-death mediated by inflammation which is known with the term of pyroptosis (125, 126). Several PRRs families are involved in inflammasomes activation, in both mice and humans, including the nucleotide-binding domain, leucine-rich repeat containing proteins (NLRs, NOD-like receptors), and absent in melanoma 2-like receptors (ALRs, AIM2-like receptors) (127). Following stimulation, the relevant NLR or ALR oligomerizes and becomes a caspase-1-activating scaffold. Inflammasomes have been linked to several auto-inflammatory and autoimmune diseases, neurodegenerative diseases (e.g., multiple sclerosis, Alzheimer's disease, Parkinson's disease), and metabolic disorders [atherosclerosis, type-2 diabetes (T2DM), obesity] (126). Inflammasomes play either causative or contributing roles in inflammatory diseases onset, and also increase the severity of the condition in response to host-derived factors (119). Many PRRs can sense metabolic signals, such as free fatty acids (FFAs) and ceramides (CERs), whose blood concentrations increase during aging. These signals activate critical inflammatory signaling cascade pathways, such as IκBα kinase/nuclear factor-κB (IKK/NF-κB), endoplasmic reticulum (ER) stress-induced unfolded protein response (UPR), and NLRP3 inflammasome. Notably, other than in immune cells, PRRs are expressed in several metabolically active tissues (liver, SKM, AT) where they prime the inflammatory cascades (128).

Besides the established role of inflammation (and age-associated low-grade inflammation) in the pathogenesis of osteoporosis (129–131), very recent findings have linked it to inflammasomes activation. PA counteracts all the molecular mechanisms involved in inflammatory signaling cascades and inflammasome complexes activation (128). In post-menopausal osteoporosis, IL-18 blood levels are increased while those of its antagonist, IL-18BP, are decreased. According to Mansoori et al., IL-18BP enhanced murine osteoblast differentiation and inhibits the activation of NLRP3 inflammasome and caspase-1, *in vitro*, and improved the metabolic and bone statuses in ovariectomized rats (a rodent model of post-menopausal OP) (132). Further evidences derived from the association of NLRP3 mutations with arthropathy and OP (133) and the SIRT1-dependent inhibition of osteogenic differentiation and enhancement adipogenic differentiation, in mesenchymal stem cells (MSC), following lipopolysaccharide (LPS)-induced NLRP3 inflammasome activation (134). It is known that bone matrix organic and inorganic components, released during high-rate bone turnover (e.g., chronic low-grade inflammation, estrogen deficiency, primary hyperparathyroidism), promote osteoclastogenesis. This process, however, was importantly reduced in Nlrp3^−/−^ cells and mice and pharmacologic inhibition of bone resorption (with bisphosphonates, e.g., zoledronic acid) attenuated inflammasome activation *in vivo*. These evidences suggest that the DAMPs-NLRP3 inflammasome axis may represent a novel mechanism supporting bone resorption (135).

PA effectively counteracts all the molecular mechanisms involved in the inflammatory signaling cascades (e.g., IKK/NF-κB, ER-UPR, inflammasomes) although the evidences about the effects of exercise on inflammasome are currently limited to NLRP3 activation and only in mouse models and in obesity. According to Ringseis, in obese mice both endurance (treadmill, 80% VO_2_max, 10 weeks) and resistance exercise (intermittent vertical holding, 10 weeks) decrease NLRP3 mRNA in AT and IL-18 in plasma (128). A number of human studies demonstrated that PA reduces plasma IL-18 levels providing the evidence for the exercise-dependent NLRP3 pathway inhibition: 12-week aerobic interval training in males and females with metabolic syndrome; 6-month aerobic training (50–85% VO_2_max) in overweight T2DM individuals; 8-week high-intensity training on a rowing ergometer (≥70% VO_2_max) in obese. In diet-induced obese rats, exercise strongly reverses TLR4 signaling and IKKβ phosphorylation in AT, SKM, and liver, suggesting a priming role for the exercise-induced inhibition of NLRP3 inflammasome. Key primers of NLRP3 activation are saturated FFA and CERs, whose circulating levels are increased in aging and metabolic dysfunctions while are decreased in response to exercise in obese animals and humans. Exercise may also reduce ER stress that primes NLRP3 activation via ROS production and NF-κB activation (128).

### Extra-Immune Systemic Inflammation and Bone Metabolism

Beside the above described prototypic adipo-myokine IL-6 with its anti-inflammatory actions (induction of IL-10 and IL-1ra and inhibition of IL-1β and TNFα), SKM and AT secrete a plethora of active molecules that act in autocrine, paracrine, and hormone-like fashion the blood concentrations of many of which have been associated with several metabolic, immune-related, and age-related pathological conditions (3). It is well-known that the post-exercise rise of circulating IL-6 is supported by SKM (136) but contrarily, chronically slightly elevated blood IL-6 are found in metabolic conditions such as metabolic syndrome and insulin resistance, obesity, and T2DM (137–139). In these cases, the main source of IL-6 is represented by the visceral AT, the liver, and the activated immune cells upon NF-κB signaling (140). Indeed, in both overweight and lean males the contribution of the AT to the circulating amount of IL-6 is mainly in the post-exercise phase (141).

In bone, IL-6 stimulates bone resorption by enhancing osteoclastogenesis/osteoclast differentiation throughout the induction of RANKL expression (142, 143) and by inducing prostaglandin E2 (PGE2) expression in osteoblasts (144–146). Bones from IL-6 transgenic mice developed osteoporosis in association with an increased number of osteoclasts and decreased osteoblasts while, on the contrary, IL-6 knock out (KO) improved the arthritis phenotype, associated with a reduced osteoclast recruitment at the erosion sites, in a murine model of arthritis (147, 148). Ovariectomy in rats, model of postmenopausal osteoporosis, decreased trabecular bone volume (TBV) and impaired hormone and inflammatory profile (decreased estradiol and calcitonin and increased bone-derived IL-1β, IL-6, and cyclooxygenase-2). Contrarily, the treadmill-exercised counterparts displayed an overall improved phenotype (149). Parallel, postmenopausal women have BMD and muscle strength correlated with soluble IL-6 (150).

Low-impact high-intensity interval training (HIIT) acutely increased bone alkaline phosphatase activity (BAP) and the expression of OPG, RANKL, and pro-inflammatory cytokines (IL-1α, IL-1β, IL-6, TNFα) while decreased bone resorption [N-terminal cross-linked telopeptide of type I collagen (NTx)] (151). In obese subjects (*n* = 173), the degree of obesity and BMD were related to IL-6 levels (in males), osteocalcin (in females), C-reactive protein (CRP), and leptin indicating that adiposity and systemic inflammation are associated with low BMD (152). In obese, a 32-week combined loading training improved muscle strength and BMD at various sites along with an improved metabolic/inflammatory status (decreased CRP, interferon (IFN)-γ, IL-6) (153).

The leukemia inhibitory factor (LIF), a myokine belonging to the IL-6 superfamily, stimulates the proliferation of satellite cell which is essential in post-injury muscle regeneration (e.g., exercise-induced muscle damage, EIMD) and SKM hypertrophy (154). It was induced, at least in term of mRNA, by acute aerobic and resistance exercises (155). In bone, LIF stimulates bone turnover, osteoblast proliferation and bone matrix deposition, and prostaglandin-induced bone resorption depending on the differentiation stage of the target cells: enhancement of differentiation in progenitors, inhibition of function (e.g., mineralization) in mature osteoblasts (156). Exercise-induced LIF acts on periosteal osteoblast in order to modulate their activity (157).

IL-7 is essential for T-cell and B-cell development (136) but it is also expressed by the contracting muscles where it acts paracrinally to induce migration in satellite cells (158). As such, IL-7 mediates the oestrogens deficiency-induced bone loss: it induces RANKL and TNFα expression in T-cells (159) and, hence, it activates mature osteoclast and stimulates the progenitors differentiation (160) as also observed *in vivo* in mice following systemic administration (161). It also promotes survival and differentiation of dendritic cells, B220+ subset, into osteoclasts (162). However, despite their beneficial effects on bone, exercise and training seem to increase the SKM expression of IL-7. Indeed, plasma IL-7 has been found to be induced, in elite female soccer players, after a 90-min soccer games (163), 30-min post resistance exercise but not after 12 weeks of resistance training (164), while the mRNA expression level was induced in SKM by 11-week long strength training (158). Therefore, as IL-6, the biological significance of SKM- and IS-derived IL-7 resides into basal-to-peak ratio (3).

In addition, IL-15 is considered an exercise-related myokine although the effect of exercise on its expression and secretion by the SKM are not well-understood. Current evidences account for a role of IL-15 in the first phase of adaptation to exercise since it has been found upregulated (in muscle and in blood) in inactive/normally active subjects following an acute bout of exercise but not following the completion of a training program (165). Indeed, IL-15 mRNA expression in SKM was not affected by a 3-h treadmill run in trained subjects (marathon runners) (166) as well as in healthy physically active men after 3-h cycling (167). However, despite no changes in plasma levels after 12-week endurance training, the protein content in SKM was increased (167). Increased blood IL-15 was found after a 30-min run on a treadmill at 70% of maximum heart rate in untrained healthy young men (168), acute resistance exercise in young healthy inactive subjects but not after 10-week chronic training (169) as well as SKM mRNA, in healthy normally active men, after an heavy bout resistance exercise (170). IL-15 is a powerful inducer of TNF-α expression, and hence of RANKL, in osteoblasts and stromal cells, resulting in enhanced osteoclastogenesis. Furthermore, in rat bone marrow cultures, it stimulates pre-osteoclasts differentiation independently from TNF-α (171). IL-15 acts synergistically with RANKL in osteoclastogenesis by activating ERK (172). These data suggest that IL-15 positively regulate osteoclastogenesis (173).

Myostatin, is a member of the transforming growth factor beta (TGF-β) superfamily [also known as growth-differentiation factor (GDF)-8], negatively regulates SKM hypertrophy and hyperplasia (174) and it may cause SKM mass loss during aging (sarcopenia) and with metabolic and inflammatory conditions (175, 176). As such it is negatively regulated by PA (acute endurance (177), acute resistance (178–180), chronic (6-month) aerobic training in overweight and obese men (181). The recovery strategy seems to have in determining the net effect of exercise (182). Different factors act as antagonist of myostatin and among them follistatin (FST) (183), follistatin-like 1 (FSTL1) (184), and decorin (185). Acute resistance exercise did not affect follistatin mRNA expression in SKM from lean young and old men (180) while it had enhancing effects in postmenopausal women (179). Decorin mRNA expression in SKM was induced by chronic combined strength and endurance training (186) but also by acute endurance exercise (187) and, in terms of plasma levels, by acute resistance exercise (186). FSTL1 plasma levels were increased in young healthy men after acute endurance exercise (188) while chronic strength training induced mRNA expression in SKM (189). Myostatin has direct effects on osteoclastogenesis (190). Indeed, osteoclasts number on trabecular bone surfaces is increased in unloading conditions in both wild-type and myostatin KO mice (191), however myostatin deficiency suppresses subperiosteal resorption with unloading, suggesting that at least a part of the effects myostatin on osteoclasts are localized to the muscle-bone interface (192). Wnt-independent bone resorption consequent to strong endurance effort (e.g., ultramarathon) has been associated to increased myostatin and decreased FST (193). FST and its related factors (FSTL1, FSTL3, decorin) are induced by exercise and their importance in bone and muscle development is evidenced in the severe phenotypes consequent to their mutation. Fstl3^−/−^ mice experienced frequent fractures together with the loss of mechanosensitivity which led to the loss of bone gain and Sost response to exercise. Importantly, a decreased FSTL3 expression is associated with aging (176). FST is induced by hyper-gravity and inhibited by microgravity (194).

Although its indisputable role as a myokine (195), brain-derived neurotrophic factor (BDNF) is mostly expressed in the brain, as is its receptor (196, 197), indeed 70–80% of the circulating protein origins from brain (9). BDNF plasma levels are raised by acute endurance and high-intensity, but not low-intensity, exercise in both males and females (195, 198–200) and also in response to chronic endurance training in young adult males (201). On the other hand, 10-week chronic resistance exercise did not affect BDNF serum levels compared to physically inactive (202). Interestingly, BDNF plasma concentrations were found to be lower in young compared to middle-aged women. After high-intensity resistance exercise BDNF follows a biphasic response featured by a decrease 1 h and an increase 24 h post exercise (203). On the contrary, 12-week moderate aerobic training (nordic walking) increased circulating BDNF in middle-aged women in association with improvement in cognitive functions (204). The BDNF receptor, TrkB, is expressed by active trabecular osteoblasts, growth plate hypertrophic chondrocytes during intramembranous ossification, and in osteoblasts and endothelial cells in fracture healing site (205, 206). Mice BDNF conditional KO in brain, beside the metabolic phenotype (hyperphagia, increased abdominal AT, obesity, leptin resistance), displayed increased femur length, high BMD and BMC (207).

The chemokine MCP-1 (also known as CCL2) is the primary ligand for the CCR2 receptor, which is normally expressed on monocyte/macrophages. As such it is a key regulator of osteoclastogenesis and has a pivotal role in inflammation and tumor-induced osteolysis (208). It is also an adipo-myokine acting as chemoattractant for monocytes and T lymphocytes (209). MCP-1 expression in SKM is strongly induced by acute and chronic resistance exercise, in terms of both mRNA and protein (164, 210), in healthy young and elderly (211). Also acute endurance activities (70% VO_2_max for 40 min, high-intensity treadmill running) increase mRNA expression in SKM in lean, obese, and T2DM (164), and circulating protein in well-trained male runners subjects (212). Therefore, expression of MCP-1 seems to be influenced by the intensity of the exercise rather than the kind of activity. Moreover, MCP-1 is affected by acute exercise while its response is not affected by training in both young and elderly healthy subjects, males and females, regardless the type of activity (211, 213).

The pro-inflammatory TNFα, the prototypic early mediator of local inflammation and initiator of the acute phase response, is expressed, other than from the immune cells, also by AT and SKM. In the AT, its expression is related to the fat mass (140) while in the SKM, the mRNA expression is inhibited following training (endurance and resistance) (214, 215). The muscle expression is neither affected by the metabolic status, being comparable between lean, overweight, and T2DM, nor by acute exercise (141, 216, 217). Circulating TNFα concentrations, however, are inversely related with the amount of PA (218) but, contrarily to moderate-intensity exercise, high-intensive training causes a temporary rise in systemic inflammation (e.g., TNFα) during recovery in response to muscle damage (10) as a propaedeutic step needed for the following regeneration (219). TNF-α is a powerful stimulus for bone resorption and is strikingly implicated in inflammatory bone diseases (220). By activating NF-κB signaling, it induces osteoclast differentiation form progenitors in the presence of M-CSF and in the absence of RANKL (221) and it also enhance RANKL sensitivity in osteoclast progenitors by inducing the expression of RANK (222). TNF-α can accelerate RANKL-dependent osteoclastogenesis by activating NF-κB and AP-1 throughout TRAF2/5 and MAPKs cascades (223) and RANKL enhances TNFα-induced osteoclastogenesis via TRAF6-independent signaling (224). TNF-α stimulates osteoclastogenesis also indirectly by inducing the expression of M-CSF and RANKL stromal cells, osteoblasts, and activated T cells (161, 225).

Visfatin/NAMP (alternatively known as pre-B cell colony-enhancing factor, PEBF) (226) is synthesized as both an intracellular form, acting as nicotinamide phosphoribosyltransferase (eNAMPT) in NAD biosynthesis, and extracellular one, visfatin, mainly secreted by the visceral AT, acting as an insulin-mimetic, pro-inflammatory/immuno-modulating adipokine (227). Its circulating levels are associated with obesity/fat mass, insulin resistance (228), and the energy-bone crosstalk (57). NAMPT expression was two-fold higher in SKM of athletes compared to that found in SKM from sedentary obese, non-obese, and T2DM subjects (229). However, in obese (>30 kg/m^2^) men no difference circulating visfatin concentrations were found between subjects with high and low cardiorespiratory fitness (230). NAMPT mRNA expression and protein content in the SKM of the exercising leg was doubled compared to the non-exercising limb, of non-obese sedentary individuals after 3 weeks of one-legged endurance exercise training endurance training (231). Acute exercise, instead, affects eNAMPT/visfatin in an intensity-dependent manner: 3-h cycling at 60% VO_2_max had no effect (232) while an acute bout of high-intensity running-based anaerobic sprint exercise had an inductive effect (233). *In vitro* visfatin stimulates osteoblast proliferation through the activation of insulin-receptor (234), induces osteoblastic differentiation in association with inhibition of OC expression (235), inhibits osteoclastogenesis throughout the suppression of RANK and NF-AT pathways (236), stimulates adipogenesis in mesenchymal stem cells (MSC) throughout the induction of PPAR-γ (237). Plasma visfatin did not differ between less than moderately trained subjects and experienced ultramarathon runners but, in this latter group it was two-fold induced after a mountain ultramarathon (39). Similarly, despite the worse metabolic profile, sedentary subjects had comparable serum visfatin concentration than professional rowers (238). Patients with metabolic syndrome had higher plasma visfatin than their age-matched counterparts which was correlated with lumbar spine BMD in men (239). Furthermore, in different women population (Chinese, Iranian) visfatin independently predicted BMD (240, 241) although it was not correlated with either BMD or BMC in a cohort of adolescent female athletes, participating in different sports (242).

Adiponectin is a prototypic adipokines that increases fatty acid oxidation and glucose uptake in SKM while inhibiting hepatic gluconeogenesis (243) and its circulating levels are inversely related to BMI and adiposity (244). It has anti-inflammatory effects since it inhibits expression and secretion of TNFα in macrophages and induces the expression of IL-10 (245, 246). Although it is considered a classical adipokine, adiponectin is also expressed by the SKM (247, 248). Plasma adiponectin levels are decreased in obesity and insulin resistance but the SKM expression of its receptors (AdipoR1 and R2) is increased (249). Acute exercise has no clear effects on circulating adiponectin with researches depicting inductive (250, 251), depressing (141) or no effects (252–256), regardless the metabolic state of the subjects. Contrarily, in healthy lean and overweight and obese subjects with impaired glucose tolerance, endurance training increased plasma adiponectin levels and induced the expression of AdipoR1/R2 in SKM (257). In highly-trained professional cyclists, plasma adiponectin was increased during a 3-week stage race (38). Fatouros and co-workers, instead, reported that high-intensity, but not moderate-intensity, either acute or chronic resistance exercise increased plasma adiponectin levels (258, 259), suggest that exercise intensity is a key determinant of the regulation of adiponectin release in blood. Among the adipokines, adiponectin is the most closely associated with BMD (negative) and fracture risk (positively) regardless gender and menopausal status (260). Hence, it exerts negative effects on bone mass although it is inversely associated with fat mass, promotes insulin sensitivity, and fat oxidation. However, bone osteocalcin induces adiponectin expression in adipocytes that, in turn, improves glucose tolerance (261, 262). AdipoRs and adiponectin are expressed by osteoblasts and osteoclasts (263, 264). Current evidences suggest that adiponectin acts autocrinally/paracrinally to simulate osteoblast function, while systemic adiponectin has inhibitory effects on osteoblasts activity while enhances osteoclastogenesis (265, 266).

Leptin is another adipokine involved in the regulation of energy homeostasis (267) it is an adiposity signal that suppresses appetite. As for adiponectin, also leptin is expressed in SKM (268) but the relative contribution of AT and SKM to circulating leptin has been not fully understood (165). Leptin and leptin receptor KO mice (ob^−/−^ and db^−/−^) which are obese have also higher bone mass and intracerebroventricular infusions improved the metabolic status and reverted the high bone mass phenotype (269). Leptin effects on bone formation are mediated by the sympathetic nervous system (SNS), independently from AT, indeed by high bone mass phenotype obtained following the inhibition adrenergic signaling cannot corrected by intracerebroventricular infusion of leptin (270, 271). In strenuous exercise-induced hypogonadal women, leptin induced oestrogens that partially improved the bone phenotype (272). Circulating leptin was decreased in highly trained professional cyclists during a 3-week stage race (38) and in experienced ultramarathon runners after a mountain ultramarathon (39). However, in these runners resting levels of leptin were significantly lower than their less than moderately trained counterparts (39). Interestingly, in competing professional cyclists the decrease in leptin was associated with increased bone resorption and GluOC-to-GlaOC ratio (38). Similarly, 8-week aerobic training decreased fat mass and leptin, improved insulin sensitivity, and increased both total OC and GluOC in obese young males experienced (273). On the contrary, in competing ultramarathon runners, a comparable trend in leptin was associated with a reduced GluOC-to-GlaOC ratio (39). Finally, soluble leptin, insulin, and OC were increased by bed rest independently from resistive vibration exercises (274). These data indicate that load may regulate leptin release. Leptin mRNA expression in AT after acute endurance exercise were found either unaffected (275) or decreased in lean and overweight subjects (141). Several studies have shown a delayed (24–48 h post exercise) reduction of circulating leptin levels in healthy active men (276–279). Taken together, the current evidences suggest that exercise training decreases plasma leptin levels, while is ineffective on mRNA expression in AT (165). Weight loss in elderly obese accelerated bone turnover but PA can attenuates BMD decrease and stimulated a greater decrease in circulating leptin (280). The detrimental bony effects of leptin also depends upon its pro-inflammatory action: stimulation of neutrophil chemotaxis and phagocytic function, induction of pro-inflammatory cytokines in monocytes, and induction of T helper (Th)-1 cytokines (246). These data suggest that the exercise-dependent beneficial effects on bone may be also mediated by the exercise-dependent reduction in circulating leptin (3).

Resistin, an inflammatory marker, is positively associated with fat mass, waist circumference, and obesity-related diseases, and it causes oxidative stress and nitric oxide production downregulating, thus, determining endothelial dysfunction (281, 282). Both circulating levels and AT mRNA expression are not affected by acute endurance training in overweight and lean males (141, 253). However, the baseline training status of the subjects seems to affect the resistance exercise-dependent response to exercise with regularly training subjects experiencing a decrease over 6 months (281). Also osteoclasts, osteoblasts, and bone marrow-derived MSC express resistin, and *in vitro* it may stimulate both osteoclastogenesis and osteoblastogenesis (234, 235). Similarly to visfatin, resistin induces PPAR-γ expression in MSC and, thus, the adipogenic differentiation (237). It has been negatively associated with BMD (240, 283), although not definitively (284–286), and in postmenopause its circulating levels are doubled compared to premenopause (287). Resistin correlated positively with previous osteoporotic fractures and much more in the presence of diabetes (288).

Irisin is a newly discovered myokine released into the circulation following the cleavage, mediated by unknown proteases, of the transmembrane glycoprotein fibronectin type III domain containing 5 (FNDC5). In target cells, mainly white adipocytes, it induces the expression of the mitochondrial uncoupling protein 1 (Ucp1) that uncouples the respiratory chain from the oxidative phosphorylation: the energy derived from the oxidation of energy substrates (e.g., carbohydrates, fatty acids), and generated by the passage along electrochemical gradient of the electrons, is released as heat. This process normally occurs in the thermogenic BAT rather than in fat-storing WAT. Irisin induces a metabolic shift in the white adipocytes, namely browning, making them expressing an intermediate beige phenotype (289). Irisin is induced by exercise and its circulating levels are higher in trained, males and young subjects than in sedentary, females, and elderly, as a function of the muscle mass and the muscle activation level (290). Along with animal studies (291), researches in human have highlighted that high-intensity acute exercise (292), endurance training (293, 294), cold exposure (295), lifestyle changes as in obese children (296) and pregnant women (297) all increase blood irisin in association with an improved metabolic status. However, these results have not been always replicated and there are still doubts about its physiology (182, 291). Such discrepancies could be, at least partially, imputed to the methodological issues emerged about some commercially available immunoassays when compared to the gold standard mass spectrometry-based method (298). Irisin is also expressed by the AT and respond to PA as for its muscle counterpart (299, 300). Other than being directly induced by exercise, irisin expression is regulated by several exercise-modulated factors such as BDNF, myostatin, follistatin, TGFβ, FFAs, cytokines, betatrophin (291). Irisin could be a link between exercise and BDNF expression (289). Indeed, 30-day voluntary free running-wheel induced FNDC5 expression in mice hippocampus, which in turn into and increased expression of BDNF (301). This correlation might support the neuroprotective effects of exercise. Still, data directly evaluating the impact of exercise (especially in human), its time duration and intensity on irisin, BDNF, and cognitive function are unclear. By affecting adiposity, irisin can improve the inflammatory status and, recently, a direct relationship between irisin concentrations and inflammatory markers in metabolic syndrome has been described (302). Irisin is involved in the SKM-bone endocrine connection and it its involvement in bone mass gain in muscle disease-associated osteopenia has been proposed (303). Irisin levels have been, indeed, associated with osteoporotic fractures in postmenopausal women (304) with an inverse correlation with fat mass and PA status (305). *In vitro*, irisin promotes osteoblast differentiation while *in vivo* it induces osteoblast proliferation and differentiation, inhibits osteoclast activity, increases cortical BMD (306), and prevents muscle atrophy-induced bone loss (307).

### Adaptive Immunity Activation and Bone Metabolism

Besides the role of innate immunity, several examples exist about the interplay between bone and IS. First of all, as already stated, osteoclasts are monocytic/macrophagic origin and M-CSF, a key cytokine for this lineage is also important in osteoclast differentiation (308) and antigen-presenting cells, such as dendritic cells, retain the capability to transdifferentiate into bone-resorbing osteoclasts (309, 310). Moreover, several soluble mediators regulate osteoblasts and osteoclasts differentiation and activity (311). Interestingly, a subset of osteogenic cells, called N-cadherin-positive spindle-shaped osteoblasts are an integral part of the hematopoietic stem cell (HSC) niche and solve a key role in the maintenance of the HSC pool that gives rise to all blood and immune cells (312).

When activate, under inflammatory conditions (e.g., autoimmune diseases, inflammatory bowel diseases, periodontal infections), T- and B-lymphocytes secrete RANKL and TNFα that stimulate osteoclast differentiation and function and, therefore, bone resorption (313). However, under physiological conditions, B-cells represent an important source of the osteoclast inhibitor OPG. For instance, human tonsil B-cells secrete OPG and the activation of the CD40 costimulatory pathway on these cells, *in vitro*, further induced OPG expression (314); parallel, bone marrow B-cells contribute up to 64% of the total OPG, in mice (315). Consequently, B-cells KO mice experienced increased bone resorption rate, reduced BMD and bone mass, in association with low circulating OPG; the restoration of the B-cells pool into young B-cell prevented the bone phenotype. The ligand of CD40, CD40L, is mainly expressed by activated T-cells and the deletion of either CD40 or CD40L on T-cells caused a powerful inhibition of OPG expression in B-cells and bone loss (315). It is, thus, suggested that under physiological conditions B-cells, regulated by the T-cells co-stimulatory action, protect the skeleton by secreting OPG, while under inflammatory conditions B- and T-cells negatively affect bone metabolism by secreting RANKL and inflammatory cytokines (316).

Postmenopause-related estrogen deficiency gives an explicative example. Indeed, oestrogens mediate powerful anti-inflammatory effects and loss of estrogen causes significant proliferation of T- and B-lymphocytes (129, 317). This was demonstrated by the fact that while ovariectomized wild-type rats experienced bone loss, T-cell-deficient null mice were protected by osteoclastic bone resorption (318). RANKL is secreted by activated T but not in conditions of estrogen deficiency, in mice. Contrary, in this condition, circulating and tissue TNFα is raised (318, 319) and TNFα and TNFRI (p55) KO in mice prevented ovariectomy-induced bone loss (318). In agreement to this model, estrogen loss causes the expansion of TNFα-secreting T-cells and TNFα sustains and amplifies the RANKL-induced osteoclast-mediated bone resorption (320). IL-7 expression in several tissues anticipates T-cells expansion (321); this cytokine increases the sensitivity of T-cells to otherwise tolerogenic antigens and, hence, decreases the antigen-dependent T-cell activation threshold (320). Consequently, the differentiation of T-cells into the different T helper subsets (i.e., Th1) leads to TNFα secretion along with IFNγ, that upregulates the expression of CIITA in macrophage, a transcription factor that in turn upregulates MHCII expression and, hence, antigen presentation to T-cells, further amplifying T-cells activation (317). Also the Th17 subset is induced in this process; these cells secrete IL-17A a pro-osteoclastogenic cytokine that induces expression RANKL in osteoblasts. IL-17 expression is raised by ovariectomy (322): treatment with anti-IL-17 antibodies (323) or the IL-17 gene deletion (324) improves bone loss in ovariectomized mice. The downregulation of TGFβ is another step in this process. TGFβ is expressed in response to oestrogens and has immunosuppressive effects by inducing regulatory T-cells (Tregs) that down-regulate T-cells activation (316).

Importantly, T-cell activation-dependent ovariectomy-induced bone loss depends upon antigen stimulation. Indeed, ovariectomy in mice with silenced antigen presentation due to a mutated T-cell receptor, only responsive to chicken ovalbumin: when no antigen is presented, mice were fully protected from ovariectomy-induced bone loss while, after exogenous administration of ovalbumin (i.e., the antigen) the bone response was retained (317). Evidence suggests that, in human, these antigens are derived from the gut microbiota (325, 326) since the gut permeability is regulated by oestrogens (326).

T-cells are critical in the mechanisms of action of parathyroid Hormone (PTH) in bone (327). Chronic elevated production of PTH (hyperparathyroidism, HPT) causes skeletal and extra-skeletal diseases: primary HPT (PHPT) is associated with increased bone turnover and osteopenia (328), while secondary HPT (SHPT) is involved in the pathogenesis of age-associated osteoporosis (329). Continuous PTH infusion mimics PHPT and SHPT, while intermittent administration has pro-anabolic effects on bone (330). T-cells express PTH-1R, the functional G protein coupled PTH receptor and they may contribute to the catabolic effect of PTH, *in vivo* (327, 331). Continuous PTH treatment at doses that mimic HPT failed to induce osteoclast formation, bone resorption, and cortical bone depletion in mice deficient for T-cells (331). On the contrary, intermittent PTH stimulates Wnt10b expression in bone marrow CD8+ T-cells and activate the canonical Wnt signaling in pre-osteoblasts (332).

Currently, there are no available study depicting the effects of the exercise on the relationship between adaptive IS and bone cell function. Hence, in order to improve the use of PA as a therapy for bone loss it is necessary to increase the knowledge in this field.

## Practical Implications, Conclusions, and Perspectives

The complex net of physiological connections linking bone metabolism and IS branches in relationship with physical exercise is, now, only a little depicted. The great majority of the current knowledge concern the inflammation-mediated effects of PA while, only a few is known about the adaptive immunity-mediated effects.

Chronic PA is a powerful stimulus for bone and lifelong exercising is the most effective strategy to improve bone mass (in childhood and adolescence) and to keep bone health (in adulthood and older ages). However, there is no consensus on the best kind of PA to be prescribed at this purpose. There are evidences that sustain the effectiveness of load and impact and this is further improved when the activity is carried on in an intermittent way (54). Therefore, the role of loading is central in this discussion but, importantly, the direct effect of the applied forces onto the skeleton on the immune function are not known and this point must be developed in the next years.

## Author Contributions

GL conception, drafting, and reviewing the article. EZ drafting and reviewing the article. GB drafting and reviewing.

### Conflict of Interest Statement

The authors declare that the research was conducted in the absence of any commercial or financial relationships that could be construed as a potential conflict of interest.
